# Clinical value of magnetic resonance imaging in preoperative T staging of gastric cancer and postoperative pathological diagnosis

**DOI:** 10.3892/ol.2014.2135

**Published:** 2014-05-12

**Authors:** XIANYING HUO, KUANGSHENG YUAN, YUEXIA SHEN, MIN LI, QI WANG, LINGXIAO XING, GAOFENG SHI

**Affiliations:** 1 Department of CT/MRI, Handan Hospital of Jizhong Energy Fengfeng Group, Handan, Hebei 056002, P.R. China; 2 Department of Anesthesiology, General Hospital of Jizhong Energy Fengfeng Group, Handan, Hebei 056200, P.R. China; 3 Department of Nursing, Handan Hospital of Jizhong Energy Fengfeng Group, Handan, Hebei 056002, P.R. China; 4 Department of CT/MRI, Fourth Affiliated Hospital of Hebei Medical University, Shijiazhuang, Hebei 050000, P.R. China; 5 Department of Laboratory, Hebei Medical University, Shijiazhuang, Hebei 050000, P.R. China

**Keywords:** cancer, magnetic resonance imaging, T stage

## Abstract

The aim of the present study was to evaluate the clinical value of magnetic resonance imaging (MRI) in the preoperative T staging of gastric cancer and in the postoperative pathological diagnosis. In total, 30 patients with gastric cancer were investigated, including 19 males and 11 females (age, 50–69 years; mean age, 60 years). The preoperative depth of invasion (T stage) was evaluated according to the characteristics of the imaging performance. The evaluation results for the MRI T staging were as follows: T1 stage accuracy, 90% with a specificity of 96% and sensitivity of 60% (κ value=0.61; P<0.05); T2 stage accuracy, 86.7% with a specificity of 87.5% and sensitivity of 83.3% (κ value=0.71; P<0.05); T3 stage accuracy, 90% with a specificity and sensitivity of 90% (κ value=0.78; P<0.05); and T4 stage accuracy, 96.7% with a specificity of 100% and sensitivity of 87.5% (κ value=0.91; P<0.05). The results demonstrated that, with reference to pathological diagnosis, the MRI method exhibited high accuracy, specificity and sensitivity in determining the preoperative T stage in gastric cancer patients.

## Introduction

Gastric cancer is one of the most common types of gastrointestinal cancer in China, with notably high incidence (3.3%) and mortality (75%) rates, and is associated with a poor prognosis. Therefore, the accurate preoperative evaluation of the extent of tumor tissue infiltration may be extremely valuable.

Previously, the diagnosis of gastric cancer has predominantly been based on upper gastroenterography and gastroscopy, which directly or indirectly observes the morphology, range and pathological changes occurring on the gastric mucosal surface. These were the necessary methods to confirm the identification, location and characteristic diagnosis of gastric cancer. These methods were unable to directly reveal the stomach structure, were limited in identifying the invasion depth of the gastric wall, extra-stomach infiltration and metastasis, however, they aided to a certain extent with the qualitative diagnosis of cavity lesions (1). Recently, diagnosis has been improved by the application of the endoscopic ultrasound (EUS) and multislice computed tomography (MSCT), which clearly exhibit the location, size and shape of stomach tumors, and determine the extent of tumor invasion, lymph node metastasis and distant organ metastasis. These methods are, therefore, considered to aid with the preoperative staging of the tumor. However, in the stratified diagnosis of gastric cancer, EUS and MSCT have certain limitations, in addition, the accuracy of MSCT and EUS, with regard to detecting the depth of gastric cancer invasion and the staging, remains controversial.

With the utilization and development of magnetic resonance imaging (MRI), the accuracy of preoperative TNM Classification of Malignant Tumours (TNM) staging in gastric cancer has gradually improved and exhibited its superiority (2). Matsushita *et al* (3) previously considered that the spoiled gradient-recalled echo technique was able to display signal layers that were lower than stomach and omentum signal layers, and that the T3 stage of extraserous infiltration was likely to be expressed as disappearance of the band or hyperintense lesions that entered this band. Therefore, the use of MRI for the preoperative staging of gastric cancer has become a predominant focus in recent years. Few previous studies have analyzed the accuracy of MRI in the preoperative staging of gastric cancer and in particular, studies comparing the MRI preoperative staging of gastric cancer and pathological results are rare. In 1994, Chin *et al* (4) applied the air-barium double contrast technique to determine the histological type of gastric cancer according to the Lauren classification system. In 1999, Rossi *et al* (5) used an ordinary computed tomography (CT) warm water filling technique for the diagnostic study of a Lauren classification type of gastric cancer, which was not widely recognized due to the limitations of X-ray and ordinary CT in the diagnosis of gastric cancer. In the present study, the patients with gastric cancer underwent preoperative hypotonic water filling, MRI and dynamic contrast-enhanced and high-resolution scanning detection to determine the TNM staging. The results were compared with surgical pathology results to evaluate the accuracy, sensitivity and specificity of the MRI scans for the preoperative TNM staging of gastric cancer.

## Patients and methods

### Clinical data

In total, 30 patients who underwent surgery for gastric cancer between June 2008 and Feb 2011 at the Fourth Affiliated Hospital of Hebei Medical University (Shijiazhuang, China) were included. Prior to scanning, endoscopy was performed as the diagnostic test. The 30 patients included 19 males and 11 females (age, 50–69 years; mean age, 60 years) and there were 12 cases of gastric cardia tumor, 10 cases of gastric body tumor, four cases of gastric antrum tumor, and four cases of gastric antrum and body tumors. All of the cases were confirmed by pathology and included 15 cases of poorly differentiated adenocarcinoma, 12 cases of moderately and well-differentiated adenocarcinoma, one case of undifferentiated adenocarcinoma, one case of signet ring cell carcinoma and one case of mucinous adenocarcinoma. The 30 patients received a preoperative MRI examination one week prior to surgery and the results were assessed and identified by two experienced radiologists. The current study was conducted in accordance with the declaration of Helsinki and approved by the Ethics Committee of the Handan Hospital of Jizhong Energy Fengfeng Group (Handan, China). Written informed consent was obtained from all patients.

### Method

The Siemens 1.5T Tim Avanto MRI instrument (Siemens AG, Munich, Germany) was used. Anisodamine (654-2; 10 mg) was intramuscularly injected and 500 ml warm water was administered orally 15–30 min prior to the examination. An additional 500 ml warm water was administered prior to the patient lying on the examination table; the patients were generally in the supine position to make a major cross section. Conventional scanning with T1-weighted imaging (WI), T2WI, short inversion time inversion recovery, diffusion weighted imaging and enhanced scanning were performed. A gadopentetate dimeglumine injection (20 ml/ampule; Beijing Beilu Pharmaceutical Co., Ltd., Beijing, China) was used for the enhanced scanning and the scan range was between the top of the diaphragm and the umbilicus. Three-phase dynamic enhancement was performed in the arterial phase (25–30 sec), the venous phase (65–70 sec) and the equilibrium phase (3–4 min), following initiation of the injection. A scan voltage of 120 kV and a current of 120 mA were used.

### T stage criteria

The following 2009 Union for International Cancer Control (7^th^ edition) TNM staging of gastric cancer was adopted: T1 stage, no thickening of the gastric wall, with abnormal enhancement of the stomach lining and enhanced tissues not exceeding the intermediate layer; T2 stage, abnormal thickening of the stomach wall, while the whole outer layer of the multilayer-structure of the stomach wall is structurally integrated or the serosal surface is smooth and tidy; T3 stage, the whole stomach is infiltrated by the tumor and the external edge of the stomach wall or peripheral-stomach adipose tissue exhibits lower or irregular signal patterns or interruption; and T4 stage, structure signals of the stomach-adjacent organs are changed or an abnormal enhancement shadow of the stomach-adjacent organs appears in the enhanced scan.

### Statistical analysis

MRI diagnosis for the T stage of 30 patients with gastric cancer was compared with the postoperative pathological diagnosis. κ values were used as the index to measure the degree of consistency. If the κ value was ≥0.75, this indicated that a very satisfactory degree of consistency had been obtained. If the κ value was <0.4, this indicated that the desired consistency level was sufficient. The present study also examined κ values using the Mann-Whitney U test. P<0.05 was considered to indicate a statistically significant difference. All statistical analyses were performed using SPSS 11.0 software (SPSS, Inc., Chicago, IL, USA).

## Results

### MRI assessment of the preoperative invasion depth of gastric cancer (T stage)

**Figure 1:**
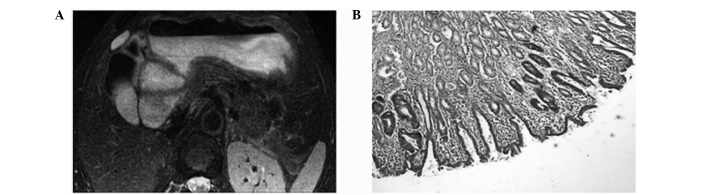
(A) Enhanced scan identified that the stomach was smooth, with no marked thickening and the gastric fat had been cleared (T1 stage). (B) Hematoxylin and eosin staining of the gastric cancer tissue (T1 stage; magnification, ×400; scale bar, 100 μm).

**Figure 2:**
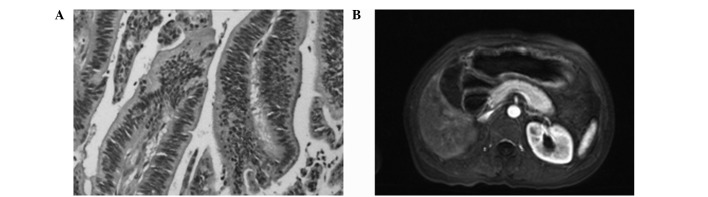
(A) Hematoxylin and eosin staining of gastric cancer tissue (T2 stage; magnification, ×400; scale bar, 80 μm). (B) Enhanced scan identified that the gastric wall was thickened and smooth, with a low-signal in the area around the stomach that was free of fat (T2 stage).

**Figure 3:**
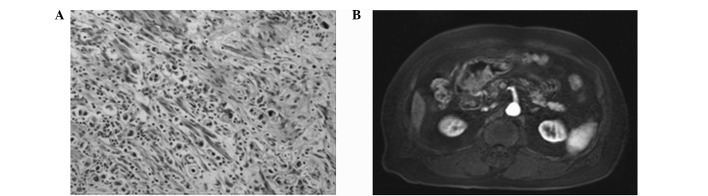
(A) Hematoxylin and eosin staining of gastric cancer tissue (T3 stage; magnification, ×400; scale bar, 50 μm). (B) Enhanced scan identified that the signal from the gastric wall was not continuous and the area around the stomach that was free of fat was narrowed by the pressure of the tumor (T3 stage).

**Figure 4:**
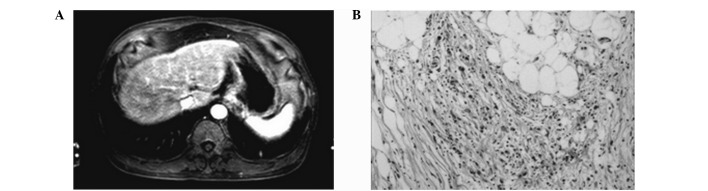
(A) Enhanced scan identified that the cardiac wall was thickened with a markedly enhanced signal (T4 stage). (B) Hematoxylin and eosin staining of gastric cancer tissue (T4 stage; magnificiation, ×400; scale bar, 100 μm).

In total, four cases of T1 stage (Fig. 1), eight cases of T2 stage (Fig. 2), 11 cases of T3 stage (Fig. 3) and seven cases of T4 stage (Fig. 4) were identified.

### Confirmation of the MRI results by postoperative pathology (T stage)

In total, five cases of T1, six cases of T2, 10 cases of T3 and eight cases of T4 stage were identified by postoperative pathology.

### Comparison between the MRI T stage results and pathological diagnosis

#### Comparison of T1 stage

MRI correctly diagnosed three cases, misdiagnosed one case and missed two cases. The MRI diagnostic accuracy of T1 stage was 90%, with a specificity of 96% and sensitivity of 60% (κ value=0.61; P<0.05; Table I).

#### Comparison of T2 stage

MRI correctly diagnosed five cases, misdiagnosed three cases (overestimating two cases and underestimating one case) and missed one case. The underestimated case was diagnosed as T3 stage due to the indistinct appearance of the external edge, while the two overestimated cases were diagnosed as T1 stage, as the thin wall induced stronger signals. The MRI diagnostic accuracy of T2 stage was 86.7%, with a specificity of 87.5% and sensitivity of 83.3% (κ value=0.71; P<0.05; Table I).

#### Comparison of T3

MRI correctly diagnosed nine cases, misdiagnosed two cases (overestimating one case and underestimating one case) and missed one case. The underestimated case was diagnosed as T4 stage due to the ambiguous expansion of the fat surrounding the lesion, while the overestimated case was diagnosed as T2 stage due to the integrated and continuous edge of the stomach wall. The MRI diagnostic accuracy, specificity and sensitivity of T3 stage were all 90% (κ value=0.78; P<0.05; Table I).

#### Comparison of T4

MRI correctly diagnosed seven cases, missed one case and no cases were misdiagnosed. The MRI diagnostic accuracy of T4 stage was 96.7%, with a specificity of 100% and sensitivity of 87.5% (κ value=0.91; P<0.05; Table I).

These results showed that the MRI preoperative T staging of gastric cancer exhibited statistical significance (P<0.001) compared with the postoperative pathological observations, particularly for the diagnosis of T3 and T4 stages. The κ values were >0.75 for the T3 and T4 stages, which reflected a relatively satisfactory degree of consistency between the two diagnostic methods.

## Discussion

Commonly, gastric cancer occurs in the gastric antrum, predominantly in the lesser curvature of the stomach, which accounts for ~75% of gastric cancers. Other types are located in the fundus (including the cardia) or gastric body and, according to the development of the gastric cancer, it may be categorized as early- or advanced-type. Early gastric cancer is defined by tumor tissues infiltrating only the lamina propria and submucosa, not the stomach muscle layer. Minute gastric carcinoma is an early gastric cancer, with a diameter of <5 mm; small gastric cancers exhibit diameters of 6–10 mm. The five-year survival rate of early gastric cancer is ≥85% and that of minute gastric cancer may be ~100%, therefore, the early diagnosis and treatment of these types of cancer is important (6). The widespread use of fiberoptic endoscopy enables the early diagnosis of gastric cancer. Furthermore, early gastric cancer may be divided into the three types; protruded, superficial and depressed. Advanced gastric cancer is defined by cancer invasion that is deeper than the submucosa, reaching the stomach muscle layer or the whole layer of the gastric wall. Prognosis worsens with the depth of carcinoma infiltration and the five-year survival rate of serosal invasion is significantly lower when compared with muscular invasion. Clinically it has been found that the majority of gastric cancers identified at the clinic were advanced. Gastric cancers may be divided into the following types by naked-eye observations: Mushroom, ulcer and infiltrating. Primary gastric cancer is an adenocarcinoma, which may also be subdivided, according to the degree of differentiation, into high and low undifferentiated types. Additionally, according to their secretion, mucinous carcinoma may be divided into signet ring cell carcinoma, nodular mucinous adenocarcinoma, colloid carcinoma and other cancer types, including squamous cell carcinoma, adenosquamous carcinoma and carcinoid. The predominant types of carcinomatosis exhibit direct diffusion, hematogenous metastasis and implantation metastases. The early symptoms of gastric cancer are not clear. As the disease progresses, stomach-related symptoms may become increasingly significant, including abdominal pain, weight loss and loss of appetite. The growing tumor may cause partial or complete obstruction of the pylorus, indigestion and vomiting, usually of gastric fluid. The appearance of carcinoma is likely to cause fecal occult blood and, following invasion into large vessels, sudden upper gastrointestinal bleeding may occur. In the late stage, an upper abdominal mass and symptoms caused by tumor metastasis, such as supraclavicular lymphadenopathy and ascites, followed by anemia, weight loss and cachexia, are likely to appear.

The pathophysiological basis of three-stage enhanced scanning is as follows. The contrast agent is intravenously injected into the elbow and flows to the aortic branch via the heart and into the systemic circulation. At this point, the concentration of the contrast agent within the aorta increases rapidly. In addition, the CT value increases rapidly and reaches it peak value (A phase). Next, the contrast agent gradually flows out of the vessel into the extravascular space (portal venous phase) and finally achieves the equilibrium phase. The gastric blood supply is rich, with the main blood supply from the left gastric artery, hepatic artery and pulmonary artery, arising from the celiac trunk. The left gastric artery supplies the lesser curvature of the stomach, the left and right gastroepiploic arteries supply the greater curvature of the stomach and the short gastric arteries supply the fundus of the stomach. Angiography of gastric cancer and microvascular studies have shown that neovascularization may be observed in the arterial and capillary phases of the majority of gastric cancer, with a rich blood supply, a high number of distorted new blood vessels and an increased vascular volume (7). Additionally, the venous phase is likely to show positive tumor staining.

Matsushita *et al* (3) previously considered the spoiled gradient-recalled echo technique to exhibit signal layers that are lower than those of the stomach and omentum, and that the T3 stage of extraserous infiltration was likely to be expressed as a disappearance of the band or as hyperintense lesions entering this band. In the present study, the delay period in the dynamic enhanced scan revealed the blurred and disappeared fat layer in the adjacent tissues and certain enhancement of the invaded tissues at the interface of the tumor. This generally lasted for ~5 min, which is marginally longer when compared with the previous literature. Therefore, the effects of enhanced scanning are more useful in the detection of early and advanced types of gastric cancer.

Originally, gastrointestinal motility led to numerous limitations for MRI, however, as new MRI technologies have emerged, it has become possible to use MRI to determine gastric cancer (3,8,9).

In normal imaging of the stomach, MRI generally exhibits between two and three or more layers of clear and normal stomach structure, with a display rate of 30–70% (3). A single-layer structure of the stomach is common, which poses a certain degree of difficulty for diagnosis. Previously, Kang *et al* (10) analyzed an *in vitro* stomach specimen by MRI; low signal stratum mucosum, low signal muscularis propria and stratum mucosum, and low or high signal submucosa were shown on T1WI. The longitudinal muscle exhibited a low signal, while the lamina propria muscle tissues exhibited high signals on T2WI. To reduce the quantity of gastrointestinal motility-related artifacts, the patients were administered 654-215 mg by an intramuscular injection and 700–1200 ml water contrast agent orally; with a subsequent Bolus injection of iohexol 100 ml to fill the stomach cavity, followed by a large volume of water to fully expand the stomach. Horizontal scanning of the gastric cancer was then performed. Kang *et al* (10) found that the gastric cancer is a three-tier structure. In addition, Wang *et al* (7) performed dynamic enhanced MRI scanning and revealed a three-layer stomach structure *in vivo,* with a significantly enhanced mucosal layer, a low signal submucosa in the middle and thickening in the muscularis and serosa. The MRI display rate of the three-layer structure was 93.3% and MSCT was 53.6% (11). This difference was found to be significantly different (P<0.05), indicating that MRI is significantly better than MSCT in the hierarchical description of gastric cancer.

MRI scanning emits no radiation to the human body and may be used to perform multifaceted and multiscan sequence horizontal scanning, which may provide a signal comparison between different imaging modalities. In addition, MRI can perform enhanced scanning repeatedly during breath-holding, without using high doses of contrast agent. MRI enables easy observation of tumor invasion depth, extent and thickening. In recent years, MRI has been widely used for analysis of the nervous and skeletal system.

The MRI horizontal scanning results of gastric cancer are as follows: Low signal intensity on T1WI and moderate but relatively low signal intensities on T2WI. When T1WI are used in combination with fat suppression sequences, the lesions appear as high signals, which indicates that high signals may be due to the suppression of fat tissue around the lesion. The moderate but relatively low signal intensity in T2WI may be due to the increased number of fibrous tissue components in gastric cancer. The mucilage ingredients present in mucinous adenocarcinoma demonstrate the lesions more clearly. Therefore, MRI horizontal scanning may be better than MSCT for the analysis of gastric cancer.

In the current study, MRI analysis of early gastric cancer (four cases of T1 stage) exhibited a high reinforced magnitude of the mucosal layer with a linear shape in the arterial phase. However, this was more evident in the parenchymal phase, in which the submucosa was continuous and complete. By contrast, the significant enhancement effect disappeared in the equilibrium phase. In the 26 advanced gastric cancer cases, a marked thickening of the inner layer was observed in the arterial phase and a gradually expanding enhancement area appeared throughout the whole lesion in the parenchymal phase.

Previously, Kang *et al* (10) reported a high display rate of the gastric submucosa, with an MRI scanning accuracy rate of >75% for T1WI. The T1WI diagnosis accuracy of gastric cancer was 77% (23/30) and increased to 87% when enhanced scanning was applied (26/30). This indicated that the scanning resolution of the enhanced MRI on the soft tissues was significantly higher than that of the horizontal scanning.

The distinction between the T2 and T3, and the T3 and T4 stages in gastric cancer has long been a focus of MSCT investigations. Serosal contour imaging of the fat around the stomach may not always be able to clearly distinguish between T2 and T3 stages and T3 and T4 stages of MSCT (9,12,13). The use of MSCT to determine T3 stage gastric cancer is more difficult when the imaging of intestinal serosa shows irregular protruding strips, thus, the application of MRI may be a solution to this problem. In the MRI images of the present study, T2 stage gastric cancer showed significant enhancement in the lag period, while the edges of the stomach wall were smooth and intact, and the outer layer showed a low signal intensity. In T3 stage gastric cancer, the fat around the lesions exhibited a film strip and showed marked enhancement following enhanced scanning. In addition, the outer layer and adjacent tissue boundaries were blurred, and an enhanced performance of the whole layer was observed. This imaging difference may be adopted for the differential diagnosis of T2 stage gastric cancer. In the determination of the T2 and T3 stages, the MRI accuracy was 86.7 and 90%, with a specificity of 87.5 and 90%, and sensitivity of 83.3 and 90%, respectively. In addition, the κ values were 0.71 and 0.78, respectively, indicating that the T3 stage was more compliant with the histological results than the T2 stage, which also complied well with the histological results.

In addition, the selection of surgical methods for the differential diagnosis of T3 and T4 stages was important. Previously, Matsushita *et al* (3,14,15) hypothesized that the spoiled gradient-recalled echo technique was able to exhibit signal layers that were lower than those for the stomach and omentum. In addition, the authors considered that the degree of extraserous infiltration could be determined by observing whether the hyperintense lesion entered the low signal band or by observing the disappearance of the low signal band. In the current study, MRI showed that dynamic contrast-enhanced scanning delayed the thickening effect at the interface of the lesions in the invaded lesion tissue during the lag period. The abovementioned MRI horizontal scanning showed blurred structures in the adjacent tissues and disappearance of the fat layer. Therefore, it was essential to enhance the scanning during the lag period (16–18), which was generally ~5 min longer than those reported in the previous literature. The effects of enhanced scanning may aid with the detection of early and advanced gastric cancer. The accuracy rates of T3 and T4 stage diagnosis compared with those of surgical pathology were 90 and 96.7%, with a specificity of 90 and 100%, and sensitivity of 90 and 87.5%, respectively. In addition, the κ values were 0.78 and 0.91, respectively, indicating that T3 and T4 staging exhibited a high degree of consistency with the pathological results. The results showed that the reason for the higher staging was due to gastric cancer combined with inflammation. Future studies concerning staging are required, as the identification of the T3 and T4 stages significantly affects the selection of the appropriate surgery (19,20), which was shown by the current study.

The correlation between the preoperative T staging of gastric cancer and postoperative histopathology is important for clinical treatment as it involves numerous factors, including whether surgical resection must be performed, selection of the surgical procedure, comprehensive treatment plans and prognosis assessment as well as other factors. The preoperative T staging of gastric cancer is also associated with the survival period and the patient quality of life, therefore, it is particularly important to obtain an early diagnosis of gastric cancer. Regular medical screening is significant to ensure that progression of the gastric cancer is not overlooked, as early diagnosis and treatment may improve patient quality of life. Furthermore, the MRI prediction of poorly differentiated stomach carcinoma is important as the malignancy of poorly differentiated gastric cancer is high and requires extensive surgical resection, which as a result, has a poor prognosis and is prone to distant metastasis. Therefore, combined modality therapy for poorly differentiated gastric cancer must be improved and all cases must be followed up.

In conclusion, MRI is valuable in the preoperative T staging of gastric cancer due to its accuracy and specificity in determining the invasion depth of gastric cancer, which may aid with guiding the selection of treatment options and avoiding unnecessary surgery. General MRI scanning has advantages and disadvantages for the T staging of gastric cancer. The disadvantages include: i) A long clinical assessment time, normally ~30–45 min; ii) a small and limited scan range; and iii) poor image quality, as dynamic enhanced scanning requires the patients to breathe repeatedly, which certain patients are unable to cope with, thus producing an unclear image. However, the continued development of MRI technology may resolve these difficulties and increase the value of adopting MRI for the preoperative staging of gastric cancer.

## Figures and Tables

**Table I:** Comparative analysis between the MRI and pathological diagnosis of the depth of invasion (T stage) in 30 gastric cancer patients.

MRI	Surgical pathology		Sensitivity^a^, % (n)	Specificity^b^, % (n)	POS prediction^c^ value, % (n)	NEG prediction^d^ value, % (n)	Accuracy^e^, % (n)	κ	P-value

	POS	NEG							
T1			60 (3/5)	96 (24/25)	75 (3/4)	92.33 (24/26)	90 (27/30)	0.61	<0.001
POS	3	1							
NEG	2	24							
T2			83.3 (5/6)	87.5 (21/24)	62.5 (5/8)	95.4 (21/22)	86.7 (26/30)	0.71	<0.001
POS	5	3							
NEG	1	21							
T3			90 (9/10)	90 (18/20)	81.8 (9/11)	94.7 (18/19)	90 (27/30)	0.78	<0.001
POS	9	2							
NEG	1	18							
T4			87.5 (7/8)	100 (22/22)	100 (7/7)	95.7 (22/23)	96.7 (29/30)	0.91	<0.001
POS	7	0							
NEG	1	22							

P-value, the possibility of occurrence of an event the size of the reaction. MRI, magnetic resonance imaging; POS, positive; NEG, negative.

a (POS surgical pathology and MRI values)/total POS surgical pathology value;

b (NEG surgical pathology and MRI values)/total NEG surgical pathology value;

c (POS surgical pathology and MRI values)/total POS MRI value;

d (NEG surgical pathology and MRI values)/total NEG MRI value;

e matched MRI and surgical pathology diagnosis/total patients.
